# Trends and Determinants of Small Birth Weight in Ethiopia: Further Analysis of Ethiopian Demographic and Health Surveys

**DOI:** 10.4314/ejhs.v31i2.13

**Published:** 2021-03

**Authors:** Ayantu Kebede, Alemi Kebede, Sena Belina, Yonas Biratu

**Affiliations:** 1 Jimma University, Institute of Health, Faculty of Public Health, Department of Epidemiology; 2 Jimma University, Institute of Health, Faculty of Public Health, Population and Family Health Department; 3 Jimma University, Institute of Health, Faculty of Health Sciences, School of Nursing and Midwifery

**Keywords:** Low Birth Weight, Small Birth Weight, Trends, Determinants, EDHS, Ethiopia

## Abstract

**Background:**

Globally, Low Birth Weight (LBW) prevalence is estimated to be 14.6%. It is a major cause of neonatal mortality in developing countries including Ethiopia. Despite extensive institution-based studies in Ethiopia, there is no comprehensive study using countrywide data. Thus, this study aimed to investigate trends and determinants of Small Birth Weight (SBW) among under-five children in Ethiopia.

**Methods:**

Under-five children data from 2000, 2005, 2011, and 2016 Ethiopian Demographic and Health Surveys (EDHS) were used. However, only 2787 children were weighed at birth and used for analysis in this study. Descriptive statistics and the logistic regression model were used to determine trends and determinants of SBW respectively.

**Results:**

The prevalence of SBW increased from 7.0% (95% CI; 3.1–10.0) to 13.2% (95% CI; 11.4–15.0) between 2000 and 2016. The odds of SBW increased by being a female child (AOR 1.50; 95% CI [1.07–2.09]), mother's with partner occupation of agriculture (AOR 1.54; 95% CI [1.05–2.26]) and mothers who did not know their partner's occupation (AOR 7.35; 95% CI [1.96–27.48]). However, infants born to mothers with primary (AOR 0.43; 95% CI [0.29–0.65]), secondary (AOR 0.30; 95% CI [0.16–0.55]) and higher (AOR 0.55; 95% CI [0.31–0.97]) educational status versus no education and grandmultiparous mothers (OR 0.39; 95% CI [0.19–0.78]) versus primiparous had lower odds of SBW.

**Conclusion:**

In Ethiopia, during the survey period, there was an increment in prevalence of SBW, and maternal related factors were significant determinants. Therefore, empowering mothers through education and improving the socioeconomic status of the household can be one strategy to reduce SBW.

## Introduction

Low Birth Weight (LBW) is defined as weight at birth of less than 2,500 grams (5.5 pounds). At birth, the weight of the baby can be low either due to preterm birth or fetal restricted intrauterine growth (1,2). A baby's birth weight can be affected by the mother's fetal growth, her diet from birth to pregnancy, and her body composition at conception. Mothers in deprived socio-economic conditions frequently have LBW infants which resulted from prolonged maternal malnutrition, infections, pregnancy complications, and physically demanding work during pregnancy (3–5).

LBW can predict the child's health throughout life and may result in a wide range of chronic diseases in future life such as ischemic heart disease, stroke, hypertension, diabetes, metabolic syndrome, malignancies, dementia, osteoarthritis, and poor cognitive development (6–9). Besides, it can also leads to reduced school performance and behavior (10,11).

Globally, in 2015 an estimated 20.5 million live births were LBW with a prevalence of 14.6%. However, a considerable variation was observed across regions and within countries on the prevalence, and 91% of the number is contributed by low- and middle-income countries, mainly Southern Asia (48%) and sub-Saharan Africa (24%) (12,13).

LBW can be caused by both direct factors related to pregnancy complications and indirect factors such as economic development (2). Studies conducted before reported that maternal age, maternal education, race, occupation, maternal weight and height, sex of the neonate, parity, smoking, inadequate antenatal care, and other factors are related to LBW (6,7,14,15).

LBW is a key indicator of progress towards global nutrition target achievement set by WHO to reduce LBW by 30% in 2025. Therefore, monitoring LBW trend is an essential component of the Global Nutrition Monitoring Framework approved by member states at the World Health Assembly (WHA) in 2015 (16,17). Although some progress was achieved between 2000 and 2015, more than doubling the progress is required to meet the target (12).

In Ethiopia, including the national survey, there is variation in prevalence of LBW between 9.9% to 28.3%(18–21). However, majority of the studies conducted were facility-based and no study reported about trends of LBW from nationally representative data. Thus, this study planned to investigate trends and determinants of small birth weight among under-five children in Ethiopia from nationally representative data. So, the finding of the study will be able to provide effective health policies to reduce infant mortality and change the directions of interventions.

## Methods and Materials

Ethiopian Demographic and Health Survey (EDHS) is a nationally representative cross-sectional survey conducted every five years. In Ethiopia, the first Demographic and Health Survey (DHS) was conducted in 2000 and consequently, in 2005, 2011, and 2016. In the survey, a stratified, two-stage cluster design was used to select a sample. Each region was stratified into urban and rural areas. In the first stage, samples of Enumeration Areas (EAs) were selected independently in each sampling stratum with probability proportional to EA size. In the second stage of selection, households per cluster were selected. Details of sampling design have been mentioned elsewhere (22,23).

Different sets of validated questionnaires containing different unit of analysis were used to collect information from different levels. Those questionnaires were eventually converted into different datasets. In this study, childrecode dataset which contained information about all children who born in the previous five years preceding the survey was used. During the four-survey period from the total births recorded, only 2787 children were weighed at birth and used for analysis in this study. The 2000, 2005, 2011, and 2016 EDHS data were used for trend analysis and the recent, 2016, EDHS data was used for determinants study. These data was obtained from the DHS data website at www.dhsprogram.com after a written request explaining the purpose of the study was communicated.

**The following operational definitions are used for this study.**

**Outcome variable:** In this study, birth weight of the infant was the outcome variable and classified into normal if weight is greater than or equal to 2500 grams, and SBW if below 2500 grams.

**Explanatory variables**: The explanatory variables included in the study were selected based on epidemiological information, prior studies, a review of the relevant published demographic studies, and the available information in the DHS datasets (7,21,24). The included variables were categorized into three: maternal obstetric factors, child factors, sociodemographic and economic factors.

Maternal obstetric factors include maternal age at first pregnancy, number of antenatal visits, history of taking iron supplements during pregnancy, parity, and history of pregnancy termination. The child factors include the child's sex, birth order, and birth interval. The sociodemographic and economic factors included maternal education and occupation, partner education and occupation, wealth index, place of residence, and region.

Maternal age at first pregnancy was categorized into four different age groups: < 20 years, 20–29 years, 30 -34 years, and ≥35 years. On EDHS, maternal and partner occupation was grouped into did not work, professional/technical/managerial, clerical, sales, agricultural, services, skilled manual, unskilled manual, others and don't know. However, in this study, we created four categories by merging professional/technical/managerial, clerical, sales, services, skilled manual, unskilled manual, and others into working (paid) group; did not work, agricultural, and don't know.

Wealth index was a composite indicator of a household's economic status which was used by DHS surveys globally (25). EDHS categorized wealth index into five different wealth quintiles. However, in this study, it was regrouped into three: poor (poorest and poor), middle (middle), and rich (richer and richest).

Parity was derived from total children ever born to the mother and categorized into (i) primiparous, (ii) multiparous (2–5), and (iii) grand multiparous (>5).

The number of Antenatal Care (ANC) visits was a continuous variable and categorized based on the minimum WHO antenatal visit recommendation: No ANC visit, 1–4 visits, and more than 4 visits.

Maternal history of taking iron supplements during pregnancy was derived from the number of days a mother took the pills and categorized into “**Yes**”, if she took at least for one day, and “**No**” if no iron supplement was used during pregnancy.

Birth order was categorized into three categories: (i) first, (ii) second or third, and (iii) fourth and above. Birth interval was categorized into (i) no birth interval for primiparous mother and (ii) less than 24 months and (iii) greater than or equal to 24 months for multiparous mother.

**Statistical analysis:** Before any statistical analysis, standard EDHS sample weight was applied on the data to account for the unequal probability of selection in the sample and nonresponse. The weighting variable used was women's individual sample weight since the study unit of analysis was women. Descriptive analysis and line graphs were performed to examine trends of SBW. Due to their known high risk of SBW, multiple births were excluded from the analysis. Region, partner education, and history of taking iron supplements during pregnancy were excluded from determinant analysis due to small cases per cell. Backward stepwise regression method was used for model building. To identify candidate variables, bivariate logistic regression at p-value <0.05 was considered, and those variables significant on bivariate analysis were further examined using multiple logistic regression. P-value <0.05 and confidence interval were used to declare the significance of the variable. Model fitness was checked by Hosmer and Lemeshow test, and the result (P value=0.447) indicated that the model is a good fit. The statistical analysis was conducted by using IBM SPSS statistics version 20. All figures and tables in the report depict weighted percentages.

**Ethics**: Ethical clearance for the EDHS was provided by the Ethiopia Health and Nutrition Research Institute (EHNRI) Review Board, the National Research Ethics Review Committee (NRERC) at the Ministry of Science and Technology, the Institutional Review Board of ICF International, and the United States Centers for Disease Control and Prevention (CDC). For this specific analysis, the data was requested; the analysis was approved and permitted.

## Results

**Population characteristics**: During the four survey periods, a total of 46,318 births were recorded in Ethiopia. Five years prior to each survey in 2000, 2005, 2011, and 2016; 12, 260, 11,163, 11,872 and 11,023 live births was born respectively. From the total births recorded, only 2787 children was weighed at birth, and information about birth weight was obtained either from maternal recall or health card. Based on the source of birth weight information, only 29 (0.2%) in 2000, 49(0.4%) in 2005, 52(0.4%) in 2011 and 109(1.0%) in 2016 was obtained from written card.

**Socio-demographic and Economic characteristics**: Variations in the occurrence of SBW were observed among nine regions and two administrative towns of Ethiopia. Across regions, there is an increment in the number of cases from time to time. In Afar, Benishangul Gumuz, and Harari regions, there is no reported SBW baby in 2000 and 2005 EDHS. During 2016, relatively more cases were reported in all regions and the three top regions with SBW were Afar (28.6%), Amhara (22.4%), and Southern Nation and Nationalities (13.2%) (Table 1).

**Table 1:** Trends of Small birth Weight among under-five children in Ethiopia by socio-demographic and economic characteristics: 2000–2016 EDHS

Variables	Trends of SBW*			
	
	2000	2005	2011	2016
	%	%	%	%
**Region**				
Tigray	5.0	0	9.8	7.6
Afar	0	0	20.0	28.6
Amhara	5.3	2.7	11.6	22.4
Oromia	10.0	20.2	12.2	13.1
Somali	10.0	15.4	13.0	11.5
Benishangul Gumuz	0	0	12.5	11.5
SNNPR†	0	20.5	6.3	13.2
Gambella	0	20.0	11.1	11.1
Harari	0	0	12.5	0
Addis Ababa	9.9	13.2	11.5	11.5
Dire Dawa	9.1	0	14.3	9.5
**Type of place of residence**				
Urban	8.1	10.2	9.0	10.8
Rural	2.0	22.7	17.3	15.4
**Wealth index**				
Poor	NA	12.4	11.2	15.8
Middle	NA	20.9	9.6	17.2
Rich	NA	12.9	10.6	11.7
**Maternal education**				
No education	11.1	27.7	13.5	18.2
primary	7.0	18.9	8.2	11.0
Secondary	6.0	7.8	11.4	7.8
Higher	0	3.7	13.0	15.3
**Maternal occupation**				
Did not work	6.9	14.0	12.7	13.3
Working (paid)	3.8	7.5	12.2	11.2
Agriculture	9.2	17.4	3.8	20.2
**Partner education**				
No education	10.0	24.3	16.9	18.3
Primary	10.3	22.9	13.1	15.3
Secondary	3.7	6.3	5.8	8.6
Higher	4.7	10.2	10.7	11.2
**Partner occupation**				
Did not work	0	12.0	13.2	14.9
Working (paid)	5.1	10.5	12.1	10.9
Agriculture	9.1	16.4	10.9	17.3
Don't know	0	0	0	40.0

* **SBW**: Small Birth Weight

† **SNNPR**: Southern Nation, Nationalities and People Representatives.

**NA**: The data for the wealth index was not available

Though there is variation in the number of SBW across maternal educational status, SBW most commonly occurs among mothers who did not attend any formal education. During 2000, 2005, 2011, and 2016 EDHS, respectively 11.1%, 27.7%, 13.5%, and 18.2% of SBW were born to mothers who did not attend any education. Similarly, 9.2%, 17.4%, and 20.2% of SBW babies were reported from mothers whose occupation was agriculture in 2000, 2005, and 2016 EDHS respectively (Table 1).

Partner educational status and occupation are the other important variables for SBW. According to EDHS 2005, 2011 and 2016 SBW is more common among mothers whose partners did not attend any education, and there is no trend between the prevalence of SBW and educational status. Regarding partner occupation, in 2000 and 2005; 9.1% and 16.4% of SBW babies respectively born to mothers whose partners' occupation was agriculture. However, surprisingly in 2016, 40% of SBW babies were born to mothers who did not know their partners' occupation (Table 1).

**Maternal obstetrics and child characteristics:** Prevalence of SBW increased from time to time based on maternal age at first birth, and the prevalence is more common among youngsters. In 2016, 14.9% of SBW occurred among mothers in the age group of 20 to 29 years (Table 2).

**Table 2:** Trends of Small birth Weight among under-five children in Ethiopia by Maternal Obstetrics and child-related characteristics: 2000–2016 EDHS

Variables	Trends of SBW			
	
	2000	2005	2011	2016
Maternal obstetric characterstics	%	%	%	%
**Number of ANC**‡ **visits**				
No ANC visit	4.3	4.9	10.1	6.9
1–4 visit	10.6	13.3	11.4	12.6
More than 4 visit	9.3	14.0	11.1	12.7
**Age at first birth**				
< 20	8.3	13.8	8.3	14.0
20–29	5.6	12.8	14.9	12.6
30–34	0	0	0	12.0
35–40	0	0	0	16.7
**Took iron supplementation during pregnancy**				
Yes	8.7	11.5	8.5	12.9
No	0	0	100	3.8
**Ever had terminated pregnancy history**				
No	7.7	12.5	10.7	13.6
Yes	4.1	19.6	12.1	6.3
**Parity**				
Primiparous	5.3	4.5	10.5	9.8
Multiparous	4.3	14.1	12.2	16.3
Grandmultiparous	13.2	15.9	9.5	7.1
**Child-related characteristics**				
**Sex of child**				
Male	6.1	9.6	9.7	10.8
Female	7.7	17.6	11.8	15.6
**Birth order**				
First	3.1	7.7	9.2	11.5
Second/third	5.5	16.8	14.9	13.6
Fourth and above	9.5	14.1	8.6	14.9
**Birth interval**				
< 24 months	6.2	8.9	12.3	18.0
≥ 24 months	8.3	17.1	10.8	13.6

‡ ANC: Antenatal Care

Parity might also affect the outcome of pregnancy. Based on 2000 and 2005 EDHS, 13.2% and 15.9% of SBW babies born to grand multiparous mothers respectively. However, in 2011 and 2016, SBW was more common among multiparous mothers (Table 2).

According to EDHS 2000 and 2016, 7.7% and 13.6% of SBW babies born to mothers who had no experience of pregnancy termination respectively. However, during 2005 and 2011 EDHS, it was more common among those who had history of pregnancy termination (Table 2).

During 2000 and 2016, across birth order, there was an increment in the prevalence of SBW. In 2000, 9.5% and in 2016, 14.9% of SBW occurred among children whose birth order was fourth or above. Yet, in 2005 and 2011, the prevalence of SBW was more common among children in second or third birth order (Table 2).

Concerning birth intervals, in 2000 and 2005 respectively, 8.3% and 17.1% of those whose birth intervals were greater than or equal to 24 months weigh low compared to those whose birth intervals were less than 24 months (6.2%) and (8.9%). However, during the 2011 and 2016 survey period, SBW was greater in those with birth intervals less than 24 months compared to those whose birth intervals were greater than or equal to 24 months (Table 2).

**Figure 1:**
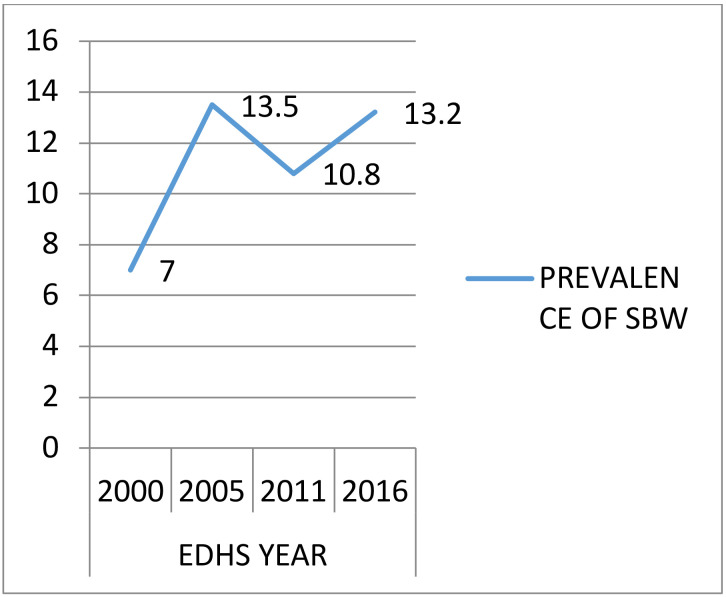
Trends of Small Birth Weight among under five children in Ethiopia: 2000–2016 EDHS

**Trends of small birth weight:** The number of SBW infants increased from 22 in 2000 to 198 in 2016. The prevalences of SBW were 7.0% (95% CI; 3.1–10.0), 13.5% (95% CI; 11.4–15.0), 10.8% (95% CI; 8.5–13.8), and 13.2% (95% CI; 11.4–15.0) in 2000, 2005, 2011 and 2016 respectively (Figure 1).

**Determinants of Small Birth Weight:** Among different variables assessed, educational status of the mother, partner occupation, parity, and sex of the infant remained significant determinants of Small Birth Weight.

Mothers who attended primary (AOR 0.43;95% CI[0.29–0.65]), secondary (AOR 0.30; 95% CI [0.16–0.55]) and higher (AOR 0.55; 95% CI [0.31–0.97]) educational level were less likely to have SBW infants compared to those who did not attend any education. Mothers whose partners' occupation was agriculture (AOR 1.54; 95% CI [1.05–2.26]) and who did not know their partners' occupation (AOR 7.35; 95% CI [1.96–27.48]) were more likely to deliver SBW infants compared to mothers whose partners participated in paid work. The odds of having SBW infant were less likely in grand multiparous mothers (AOR 0.39; 95% CI [0.19–0.78]) compared to primiparous mothers. Female infants were more likely (AOR 1.50; 95% CI [1.07–2.09]) to be SBW compared to male infants (Table 3).

**Table 3:** Bivariate and Multiple Logistic Regression model showing determinants of Small Birth Weight in Ethiopia: EDHS 2016

Factors	Birth weight		COR	P-value	AOR
	
	< 2500 g	≥2500g			
**Type of place of residence**					
Urban	67	639	1.00	0.001*	1.00
Rural	113	637	1.70 (1.23–2.35)		
**Wealth index**				0.028*	
Poor	39	211	1.53 (1.03–2.27)		
Middle	32	169	1.58 (1.03–2.42) *		
Rich	108	896	1.00		1.00
**Mothers education**				0.000*	
No education	76	349	1.00		1.00
Primary	58	494	0.54 (0.37–0.78)		0.43 (0.29–0.65) **
Secondary	18	244	0.33 (0.19–0.57)		0.30(0.16–0.55) **
Higher	28	189	0.68 (0.42–1.08)		0.55(0.31–0.97) **
**Mother occupation**				0.053*	
Not working	84	574	1.00		1.00
Working	68	571	0.81 (0.58–1.14)		
Agriculture	28	131	1.50 (0.94–2.38)		
**Partner education**				0.001	
No education	50	231	1.00		
Primary	71	412	0.79 (0.53–1.17)		
Secondary	25	270	0.42 (0.25–0.70)		
Higher	26	275	0.43 (0.26–0.71)		
Don't know	0	5	0.23(0.01–11.46)		
**Partner occupation**					
Working	84	743	1.00		1.00
Not working	7	62	0.94 (0.41–2.17)	0.000*	0.83(0.35–1.97)
Agriculture	77	382	1.77 (1.27–2.47)		1.54(1.05–2.26)**
Don't know	4	6	5.79 (1.61–20.80)		7.35(1.96–27.48)**
**Sex of the infant**				0.025*	
Male	78	667	1.00		1.00
Female	102	609	1.43 (1.04–1.95)		1.50(1.07–2.09)**
**Birth order**					
First	62	490	1.00	0.344	
Second/third	61	451	1.07 (0.74–1.56)		
Fourth or more	56	335	1.31 (0.89–1.93)		
**Birth interval**				0.108	
No birth interval	62	490	(0.65–1.29)		
< 24 months	22	99	1.59 (0.96–2.65)		
≥24 months	96	688	1.00		
**Age at first birth**				0.330	
11–19	97	600	1.00		
20–29	78	628	0.77 (0.56–1.06)		
30–34	4	43	0.53 (0.18–1.57)		
35–40	1	5	0.71 (0.04–11.90)		
**Ever had terminated pregnancy history**				0.055*	
Yes	5	82	0.45 (0.18–1.09)		0.38(0.14–1.01)
No	174	1194	1.00		1.00
**Parity**					
Primiparous	44	407	1.00		1.00
Multiparous	121	696	1.61 (1.12–2.32)	0.003*	1.40(0.93–2.11)
Grandmultipara	14	172	0.75 (0.40–1.42)		0.39(0.19–0.78)**
**ANC visit**				0.519	
No any visit	5	63	0.59 (0.23–1.53)		
1 –4 visit	78	571	1.02 (0.72–1.46)		
More than 4 visit	61	458	1.00		

* candidate variable at P value < 0.05

** significant at p value <0.05

## Discussion

LBW is a major public health problem worldwide especially in developing countries including Ethiopia. It is a major contributor to adverse pregnancy outcomes (6,15). Therefore, this study aimed to determine trends of SBW and its determinants in Ethiopia using EDHS data. Based on the study finding, there is an increment in the number of SBW from 2000 to 2016 EDHS, and educational status of the mother, partner occupation, parity, and sex of the infant were determinants of SBW.

In Ethiopia, the prevalence of SBW was increasing except slight reduction from 2005 to 2011. This national prevalence of SBW in 2016 was below that of Bangladesh (20%)(26). This might be due to the difference in the survey period considered for calculating the prevalence. In the case of Ethiopia, a sample of live births born five years preceding the survey period was considered. However, in Bangladesh, only live births in the last two years preceding the survey were included. Also, there is a difference in the number of cases for whom birth weight data were available. In Ethiopia, birth weight data were available only for 1501 cases while 78.1% of cases were not weighed at birth. However, in Bangladesh data was available for 2319 cases and on the dataset, 71% of newborns show no birth weight information.

Educational status was one of SBW determinants. In this study, mothers who attended primary, secondary, and higher educational levels less likely deliver SBW babies compared to illiterate mothers. This finding is consistent with a study conducted in Cambodia using DHS data, further analysis of 2011 EDHS, and cohort studies conducted in southern Ethiopia (27–29). Having a higher educational level had a protective effect in a way that educated women have a higher possibility of making household decisions and have access to a nutritious diet during pregnancy that would, in turn, improve their body's composition which influences infant weight. Also, they can comply with nutritional counseling during ANC visits since they may be economically stable than women who did not attend any formal education.

Besides, in developing countries deprivation of socio-economic status including educational status, occupation and others are risk factors for SBW (2,7,30). This finding implies that uneducated mothers are the most disadvantageous group both in exposure to information and ANC attendance which is crucial for being informed about different maternal cares during pregnancy and improved neonatal outcomes(31,32).

In this study, partner occupation is the other determinants for SBW. Mothers who do not know their partners' occupation and those whose partner occupation was agriculture were more likely to experience SBW. Evidence suggests that individual-level socioeconomic status (SES) is a risk factor for Small for Gestation Age (SGA) births, and low SES influence the risk of SGA birth due to residential, lifestyle, or occupational-related exposure to harmful agents (33,34). Available evidence indicates the mechanisms by which partner occupational exposure affects birth outcomes. The first is partner exposure leads to maternal exposure, and the effects will happen. The second mechanism is partner exposure leads to alteration in a germ cell line that leads to either increased infertility or abnormality in conception (35). Among the different effects of toxic chemicals, lead exposure is associated with infertility, stillbirths, and spontaneous abortions (36). However, from this specific study, we cannot be able to assess occupational-related toxic chemical exposure.

Newborn sex had gestational age-independent effects on birth weight, and female neonates had lower average birth weights than male neonates at each specific week of gestation (37,38). Similarly, in this study, female infants were more likely to experience SBW than male infants. This finding is consistent with studies conducted in Northern Ethiopia, Sidama Zone, and a systematic review conducted in Ethiopia (14,29,39). This might be due to the effect of the Y chromosome on the weight of the male fetus which needs further genetic evaluation of the pregnant mothers' population (40). Besides, it might also be due to greater lean body mass and less body fat seen in male newborns than in females, possibly due to the effects of fetal testosterone production (41). However, this needs biological investigation to identify the effects of neonate sex on birth weight.

Maternal parity is one of the well-known predictors of infant birth weight; the lowest birth weights were found in infants born to primiparous women (42). Also, in this study, parity was associated with birth weight; grand multiparous mothers were less likely to deliver SBW babies compared to primiparous mothers. This might be due to higher risks of obstetric complications such as gestational diabetes and gestational hypertensive disorders associated with grand multiparity which lead to fetal macrosomia rather than SBW (43,44).

Generally, in Ethiopia, SBW prevalence is increasing and maternal education, partner occupation, parity, and sex of the baby were determinants for SBW. Therefore, there is a need to empower mothers through education which might indirectly contribute to the reduction of SBW by improving household economic status.

As a limitation, only factors that were found on the data were retrieved and the analysis was not controlled for other genetic or biological factors. Thus, given that the etiology of SBW is complex, further research is required to address the interaction of genetic or biological factors with social and environmental factors.

In addition, possible biases like social desirability bias due to sensitiveness of the issues, recall bias due to longer recall time and measurement bias because of obtaining birth weight data from maternal subjective evaluation during the survey might affect the study finding.
